# AutoRet: A Self-Supervised Spatial Recurrent Network for Content-Based Image Retrieval

**DOI:** 10.3390/s22062188

**Published:** 2022-03-11

**Authors:** Muhammad Mostafa Monowar, Md. Abdul Hamid, Abu Quwsar Ohi, Madini O. Alassafi, M. F. Mridha

**Affiliations:** 1 Department of Information Technology, Faculty of Computing & Information Technology, King Abdulaziz University, Jeddah 21589, Saudi Arabia; mabdulhamid1@kau.edu.sa (M.A.H.); malasafi@kau.edu.sa (M.O.A.); 2 Department of Computer Science & Engineering, Bangladesh University of Business & Technology, Dhaka 1216, Bangladesh; quwsarohi@bubt.edu.bd; 3 Department of Computer Science, American International University-Bangladesh, Dhaka 1229, Bangladesh; firoz.mridha@aiub.edu

**Keywords:** deep learning, image retrieval, self-learning, convolutional neural network

## Abstract

Image retrieval techniques are becoming famous due to the vast availability of multimedia data. The present image retrieval system performs excellently on labeled data. However, often, data labeling becomes costly and sometimes impossible. Therefore, self-supervised and unsupervised learning strategies are currently becoming illustrious. Most of the self/unsupervised strategies are sensitive to the number of classes and can not mix labeled data on availability. In this paper, we introduce AutoRet, a deep convolutional neural network (DCNN) based self-supervised image retrieval system. The system is trained on pairwise constraints. Therefore, it can work in self-supervision and can also be trained on a partially labeled dataset. The overall strategy includes a DCNN that extracts embeddings from multiple patches of images. Further, the embeddings are fused for quality information used for the image retrieval process. The method is benchmarked with three different datasets. From the overall benchmark, it is evident that the proposed method works better in a self-supervised manner. In addition, the evaluation exhibits the proposed method’s performance to be highly convincing while a small portion of labeled data are mixed on availability.

## 1. Introduction

Due to the explosion of smartphones and social media, the quantity of image-based information is rapidly increasing. Like how people search for information using textual queries, the demand and usage of image-based queries are also accelerating. Image similarity search is a kind of image retrieval policy that searches images based on a given query image. The similarity of images can be determined based on various aspects, such as color [1], texture [2], shape [3], and structure [4]. As such aspects are the general content of an image, image-based similarity search strategies are termed content-based image retrieval (CBIR) [5] systems.

CBIR is currently dominating due to the heavy requirement of image-based information retrieval systems. As a result, CBIR systems are also designed to be domain-specific. Face retrieval [6] systems query for similar facial images for a given query image. Product retrieval [7] systems can identify users’ cherished products from online shopping. Cloth retrieval [8,9] systems can help consumers to identify their required product. Medical image retrieval [10] systems help diagnosis to be easy and accurate.

In contrast to such domain-specific image retrieval systems, general-purpose image retrieval systems explore the relationship of a general, unbiased dataset. Presently, search engines inherit the usefulness of CBIR systems for querying similar images. CBIR systems have two major components, understanding image content and finding similar images (based on image content) for a query image. The challenge of CBIR systems is that they still struggle with accuracy for larger datasets [11]. Moreover, CBIR systems require a vast amount of labeled data, which is time-consuming, expensive, and almost impossible to construct. Therefore, current CBIR systems are being widely exploited without the necessity of extensive data labeling.

The necessity of data labeling for training CBIR systems can be reduced in numerous approaches. Unsupervised learning [12], semi-supervised learning [13], and self-supervised [14] learning strategies are currently being inherited in CBIR systems. Amongst the various learning strategies, self-supervised learning is gaining popularity in multiple domains due to its robustness. This paper introduces a general-purpose image retrieval system based on self-supervised learning.

Semi-supervised strategies can be trained with scarce labeled data compared to general supervised architectures. In contrast, unsupervised learning strategies work with input data with no labels. Self-supervised learning strategies are trained with pseudo-labeled data. The pseudo-labels are generated based on assumptions or augmentations, and it does not require the dataset to be labeled [15]. A self-supervised model is similar to an unsupervised model as they need no labeled data, but the learning of the learner is conducted on specific data distributions.

The current semi-supervised systems are limited to hash-based retrieval methods. Hash-based retrieval methods use DCNN as a hash function to generate a binary representation for a given image. The objective of the DCNN hash function is to map closer binary representation for similar images. Although hashing methods are popular, they hardly have feature restoration and representation capability, focusing on hamming distance relationships [16]. Hence, the generated hash might not be better generalized for unknown data. The performance of binary-hash methods is dependent on the number of output bits. Therefore, hash methods with a finite number of bits will produce a limited representation, although the number of bits can be increased [17]. Some self-supervised algorithms are sensitive to the number of classes in pre-training, which is a limitation to their application on fully-unlabeled unknown datasets. Consequently, most algorithms focus on self/semi/un-supervised learning strategies, neglecting the process of a partially labeled dataset. Hence, most image retrieval systems are incapable of using the advantage of partially labeled datasets [18].

This paper introduces a self-supervised general-purpose image retrieval system with some advantages. Firstly, the training of the self-supervised method can be executed on both labeled and partially labeled datasets. Secondly, the approach performs image quantization based on DCNN architectures. Therefore, the generated embeddings are better generalized than binary hashing based on feature representation and restoration [11]. Finally, the proposed model is independent of hashing, and it is trained on deep metric learning [19]. Therefore, the proposed approach suggests distinct steps compared to the present research strategies.

The overall contribution of the paper includes:

- We introduce a CBIR system named AutoRet, which can be trained in self-supervised and can be integrated with labeled data as well.
- We utilize a recurrent network-based solution to fuse local descriptors of a single image for better performance.
- We introduce spatial polling strategies to extract resolution-independent and high field-of-view feature extraction policy in the image-retrieval system, which are usually observed in object detection and segmentation systems, respectively.
- We evaluate our model with different image retrieval techniques involving self/un-supervised strategies and validate that AutoRet performs better in all scenarios.

Section 2 highlights some of the works conducted in the image retrieval field. Section 3 introduces the AutoRet model along with architectural and training philosophies. Section 4 provides statistics of datasets used in training, explains the metrics of evaluation, and finally presents a performance benchmark. Finally, Section 5 concludes the paper.

## 2. Related Work

In any domain of artificial intelligence, supervised learning is undoubtedly accurate and robust. However, labeling a huge amount of data is almost near to impossible when thinking of production-level technology. Therefore most of the current research background is moving towards unsupervised [20] and self-supervised [14] methods. As our concern is based on models based on self-supervision, we opt-out supervised learning strategies from this review.

In the case of training DCNN models with scarce data, augmentation can effectively extend the performance of DCNN classifiers, avoid overtraining [21], and reduce the possibility of visual attack [22]. Various mathematical models can augment models, which can generate close to real signals and images [23,24]. Consequently, the performance of a self-supervised algorithm can be boosted by the proper implementation of training policy [25]. Some self-supervised architectures use appropriate data augmentation policies for generating pseudo labels [26], resulting in achieving better performance.

The present CBIR systems are largely based on hashing strategies [20,27]. Hashing methods compress images into hash codes, where the similarity search is done using hamming distance. Convolutional Neural Network (CNN) based hashing methods have gained popularity in recent years [28]. Unsupervised hashing methods have also been introduced to learn binary embeddings from images [29]. Further, hashing mechanisms have also been introduced in self-supervised learning strategies [18]. Graph convolutional neural networks (GCNN) establish graph relationships to find the similarity of images using hash embeddings [30]. Although GCNN generates better performance, the memory complexity of such systems are often high. Therefore, GCNN is difficult to implement on large datasets. Moreover, without a considerable number of relationships in a dense graph structure, it can often generate low-quality binary codes.

Although hashing is a powerful concept dominating the current investigation of CBIR, hashing methods lack proper feature representation. Therefore, generative adversarial networks (GAN) have been investigated to increase the feature representation of hash-based retrieval systems [31]; although most of them fail to preserve the similarity relationship of images, resulting in inadequate performance. In the concept of feature restoration, hashing methods require higher bits to adequately encode and decode a given image [11]. In contrast, quantization methods [32] are better in representing image semantics on an embedding space. Self-supervised algorithms are generally constructed using deep hash-based neural networks. Most deep hash-based algorithms [33,34] firstly generate embeddings from images. Further, the embeddings are used for constructing binary codes. Although DNN is powerful, it struggles to preserve the nearest neighbor relationship in the binary representation. In contrast, deep metric-based algorithms try to solve the challenge of nearest neighbor relationships by maintaining a pairwise/triplet loss. Moreover, deep metric learning strategies are similar to quantization, where the quantizer is a DCNN architecture.

Deep metric learning is widely conducted using a siamese network [35] trained based on triplet [36] or pairwise [32] loss. Deep metric learning has also gained popularity as it can learn the semantic relation of images based on pairwise similarity. Therefore, attempts have been made to adjust the loss strategy for the image retrieval process [37]. However, although adjusting the loss strategy slightly improves the performance, the challenge lies in fusing local descriptors from images for better context similarity.

To identify the context from a given image, local feature aggregation proved to be promising [38]. Local feature aggregation extracts information from a series of local regions from a given input image. Further, the local region representations are aggregated and computed to generate a final image embedding. Such aggregations are done in numerous concepts in which different pooling mechanisms are introduced. Feature pooling [39] can extract specific features from a given patch from an input image. The architecture uses CNN as local patch descriptors, and the CNN is combined with a feature pooling strategy. Further, features of each patch are again placed in a grid, and CNN is used to aggregate the local descriptors. The problem with the architecture is that the local descriptors are not resolution-independent.

Local-descriptor based architectures mostly introduce new aggregation and pooling techniques than investigating patch-based feature extraction techniques. Selective convolutional descriptor aggregation [40], sum-pooled convolutional [41], part-based weighting aggregation [42], NetVLAD [43] are some of the examples of local-descriptor feature aggregation techniques. Such techniques are either feature-centric or aggregation-centric. Therefore, in most cases, either the rich features are poorly aggregated, or the shallow features are strongly aggregated.

In contrast to the other local descriptors, the proposed AutoRet focuses on both feature extraction and feature aggregation. AutoRet extracts local features from the 3×3 patches from the input image. Moreover, as the other local descriptors miss the resolution-independent features, AutoRet focuses on such concern by using Spatial Pyramid Convolution (SPP) [44]. Moreover, to firmly increase the range of feature extraction for a given patch, Atrous Spatial Pyramid Convolution (ASPP) [45] is used. Both SPP and ASPP are used for object detection and segmentation purposes, designed for pinpointing object features from a given input.

Consequently, AutoRet is trained based on deep metric learning. Deep metric learning solves the problem of complex data connectivity issues for self-supervised training [14]. Self-supervised learning retrieval systems often apply clustering to generate pseudo labels [20,46]. Therefore, some self- supervised retrieval systems require a pre-defined number of classes [46,47]. In contrast, AutoRet does not require any pre-defined number of classes. AutoRet specifically implements the AutoEmbedder [48] strategy for training the embedding model. AutoEmbedder approach can work in a self-supervision [49], which can be also mixed with labeled data. Therefore, the proposed algorithm can be applied to partially labeled data.

## 3. Methodology

**Figure 1:**
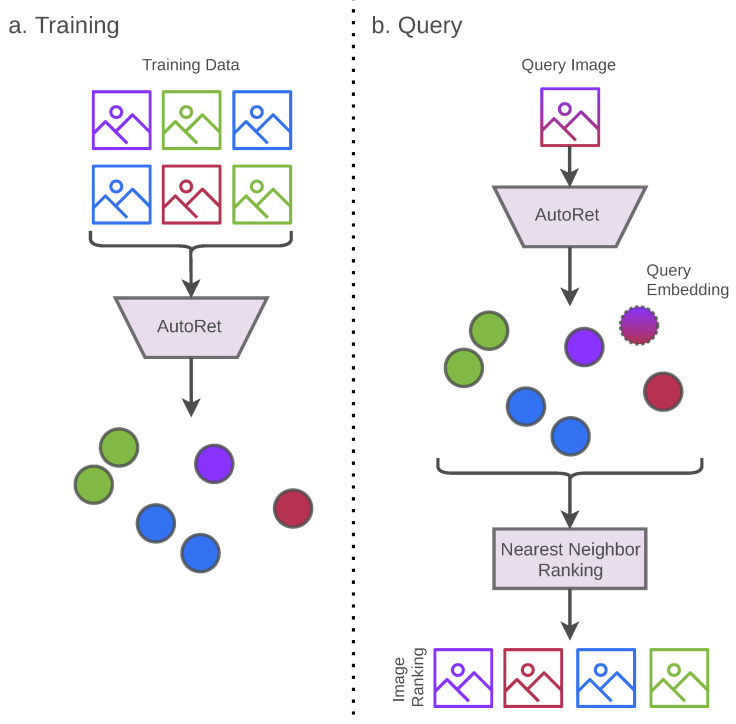
The training policy of AutoRet only includes DCNN architecture using the AutoEmbedder framework (illustrated in (**a**)). After completing the training, the trained DCNN architecture is directly used to generate image embeddings. The image embeddings of the query image and retrievable images are stored. Finally, a similarity search is conducted using a nearest neighbor algorithm based on the query image’s embedding (illustrated in (**b**)).

The general structure of the image retrieval system contains two components: (a) content-based embedding system and (b) finding similar images using the nearest neighbor algorithm. Firstly, the embedding system is trained without any label requirements for a given set of images. After complete training, the embedding system generates a content-based embedding map for the given set of images. Consequently, after completing the training, the embedding system can process any query image by generating contextual embeddings. Then a similarity ranking for the given query image can be processed by the nearest neighbor algorithm. Figure 1 illustrates each of the scenarios of the overall process.

The embedding system is the main focus of the research work. The embedding system is trained based on self-supervision. The training strategy of the embedding system is elaborated in Section 3.1. Section 3.2 explains the basic architecture of the embedding model, built using DCNN.

### 3.1. Self-Supervision through AutoEmbedder

The retrieval system is trained using Autoembedder architecture so that it can generate embeddings based on similarity. Generally, Autoembdder architecture is trained based on pairwise constraints. The policy is based on a siamese network that can be defined as follows,

(1) $$S\left(x,x^{\prime }\right)=ReLU(∥E_{\phi }\left(x\right)-E_{\phi }\left(x^{\prime }\right)∥,\alpha )=R_{\leq \alpha }^{+}$$

The ReLU(·,·) function used in Equation (1) is a thresholded ReLU function, such that,

(2) $$ReLU\left(x,\alpha \right)=\left\{\begin{array}{cc} x & \mathrm{if}\;0\leq x<\alpha \\ \alpha & \mathrm{if}\;x\geq \alpha \end{array}\right.$$

In Equation (1), the S(·,·) is a siamese network receiving a pair of input data *x* and $x^{\prime }$. An embedding model is indicated by $E_{\phi }$, which generates embedding for a given input image. The architecture of the embedding model ($E_{\phi }$) is explained in Section 3.2.

The Autoembedder strategy involves training embedding models based on the pairwise constraint. For a given training batch, half of the pairwise data contains similar image pairs, whereas the other half contains non-similar image pairs. The training target is to produce closer embeddings for a given pair of similar images. Therefore, the euclidean distance would be close to zero for a pair of a similar image. In contrast, for non-similar image pairs, the target is to produce embeddings at a minimum distance of α.

The training policy based on similarity is generated based on randomization and augmentation. Such idea of training is termed as self-supervised learning, where each piece of data is given a pseudo label or trained based on random augmentation [15]. Autoembedder strategy has already been explored for training using pseudo labels [49].

**Figure 2:**
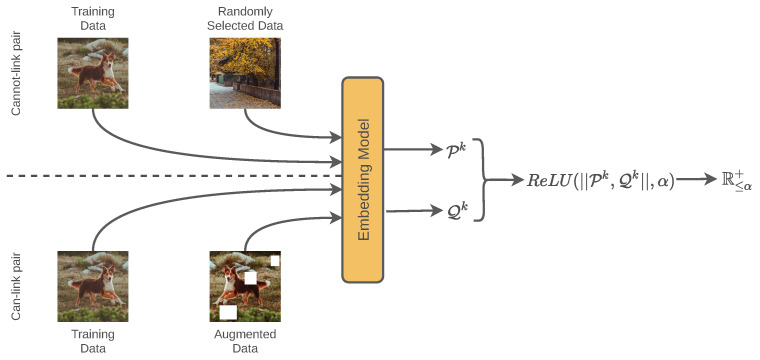
The AutoEmbedder framework generates a twin network for a given model. The objective is to calibrate the euclidean distance of the output pair of the twin network based on the image similarity and dissimilarity. For similar and dissimilar images, the output distance of the twin network should be 0 and α, respectively.

Figure 2 explains the self-supervised training process. The data selection process of the self-supervised strategy can be explained by the following two points:

- Can-link pair: For a given pair of similar images (containing equivalent content), the embedding system should generate closer embeddings-pairs. Image pairs with such a relationship are defined as can-link pairs. Half of the training data are randomly selected to generate a can-link pair with similar image pairs. If the data labels are unknown, a can-link pair can be generated using the raw image and an augmented version of that image. For augmentation, basic types of augmentation techniques, shear, random contrast/brightness, random crop, rotate, flip, noise is used.
- Cannot-link pair: For a given pair of dissimilar images (containing different content), the embedding system should generate distant embedding points. Image pairs with no content relationship are defined as cannot-link pairs. Half of the training data are randomly selected to generate a cannot-link pair representing dissimilar image pairs. For a given image, another randomly selected image is used for generating a cannot-link pair.

In the training strategy, the cannot-link pair can be erroneous, as a randomly selected image can often be of a similar class. If the number of errors in the cannot-link is huge, it would be impossible for the embedding network to converge to its optimal. Let us consider a dataset *D* consisting of $N_{c}$ classes where each class contains a uniform number of data $N_{p}$. The probability of selecting an erroneous pair ($S_{e}$) is,

(3) $$\begin{array}{cc} S_{e} & =\frac{N_{c}\times P\left(N_{p},2\right)}{P(|D|,2)}\\ \; & =\frac{N_{c}\times N_{p}\times \left(N_{p}-1\right)}{\left|D|\times (|D|-1\right)}\\ \; & \approx \frac{N_{c}\times N_{p}^{2}}{{\left|D\right|}^{2}}\\ \; & \approx \frac{N_{c}\times N_{p}^{2}}{{\left(N_{c}\times N_{p}\right)}^{2}}\\ \; & \approx \frac{1}{N_{c}}\\ \; & and,\;S_{e}<\frac{1}{N_{c}}\;\;\left[N_{c}>1\right] \end{array}$$

Therefore, for any dataset containing multiple classes, the value of selecting erroneous cannot-link pairs is always less than the correctly chosen cannot-link pairs. Hence, it can be concluded that if the function S(·,·) converges to a minimal loss value, it can adequately separate cannot-link class pairs.

### 3.2. Spatial Recurrent Network

The spatial recurrent network (SRN) is a combination of CNN and RNN used for generating embeddings from an input image. The SRN network consists of two components: (a) recurrent patching and (b) spatial network. Both components are elaborated sequentially in the following sections.

#### 3.2.1. Recurrent Patching

**Figure 3:**
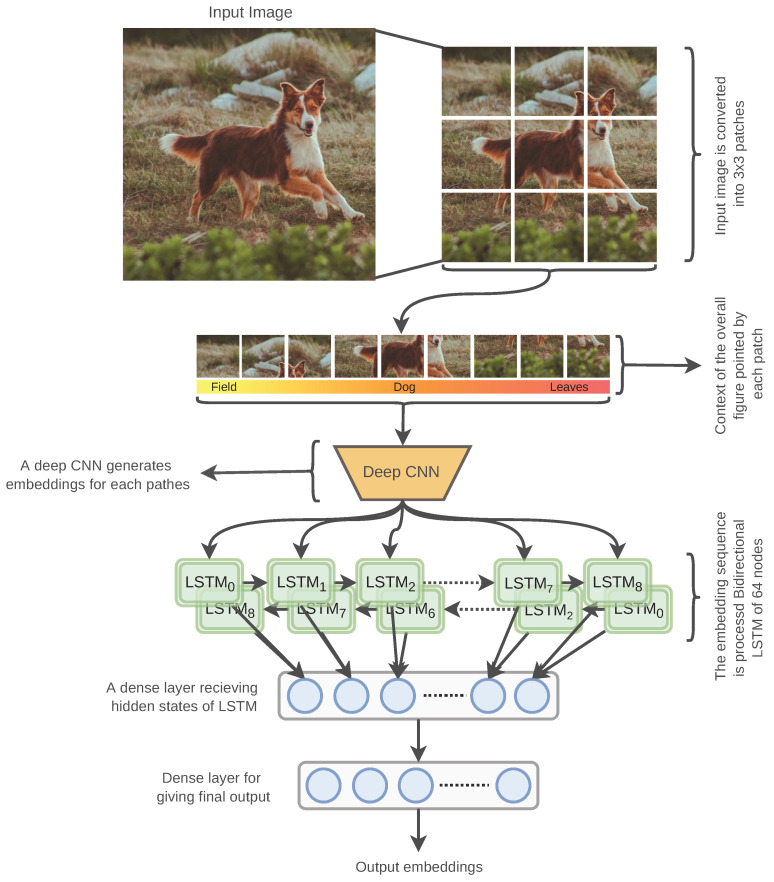
The figure illustrates the recurrent patching for generating local descriptors using DCNN architecture. A given image is firstly patched, and each patch is fed to the DCNN to generate embeddings for each patch. The embeddings are condensed using a bi-directional LSTM layer of 64 nodes. The condensed features are passed into dense layers for generating final embeddings.

The objective of the overall SRN is to not only identify the content of an input image but also to understand the underneath context of the given image. We conceptualize the context of a given image by identifying the surrounding objects of an image. In general, DCNN classifier architectures focus on finding specific contents of an image for object identification. Figure 3 illustrates the processing and architecture of the SRN.

To identify a set of contents from an image, the input image is split into 3 × 3 patches. Each patch is passed through a DCNN architecture with spatial pooling to produce higher-order features. As a DCNN architecture can output content information for a given image, we can imply that the output produced for each patch also includes content information. Therefore, merging the embeddings of each patch would integrate the patch-specific content information. A single layer of bi-directional long short-term memory (Bi-LSTM) is used to merge the path embeddings. Each of the Bi-LSTM nodes passes the hidden states to the next timestep LSTM and to the following dense layer. Finally, the dense layer is followed by another dense layer generating the final embeddings of the model. The dimension of the last dense layer controls the final output dimension of the AutoRet architecture.

#### 3.2.2. Spatial Network

The DCNN model of the retrieval system contains a general pipeline that may contain any of the present classification systems as a baseline. Any of the current adequate performing DCNN baselines can be used as a backbone in the spatial network. However, we use a pre-trained baseline model for better and fast convergence of the overall model. The objective of the DCNN model is to produce content-based embeddings as an output, which will be further integrated by the recurrent layer. As the model is specifically focusing on content features for each patch, more rich features can be captured by using the SPP method. SPP method is often observed in popular object detection mechanisms.

One of the challenges of object identification/detection is to recognize an object or a part of an object by a resolution-independent feature extraction policy. SPP deals with identifying resolution-independent features from a given input image. For each given patch, it is necessary to identify a subset of features, which aggregately help the model to determine a final object. SPP would assist to identify local resolution-independent features that would help identify global features.

Parallelly, ASPP probes each of the pixels of an image to condense the surrounding features. ASPP guarantees a better field of view and enables to identify proper contextual features for a given input patch. Therefore, features of bigger size objects can be easily extracted. Moreover, a wide pixel relation can also help to distinguish between foreground and background features of images.

**Figure 4:**
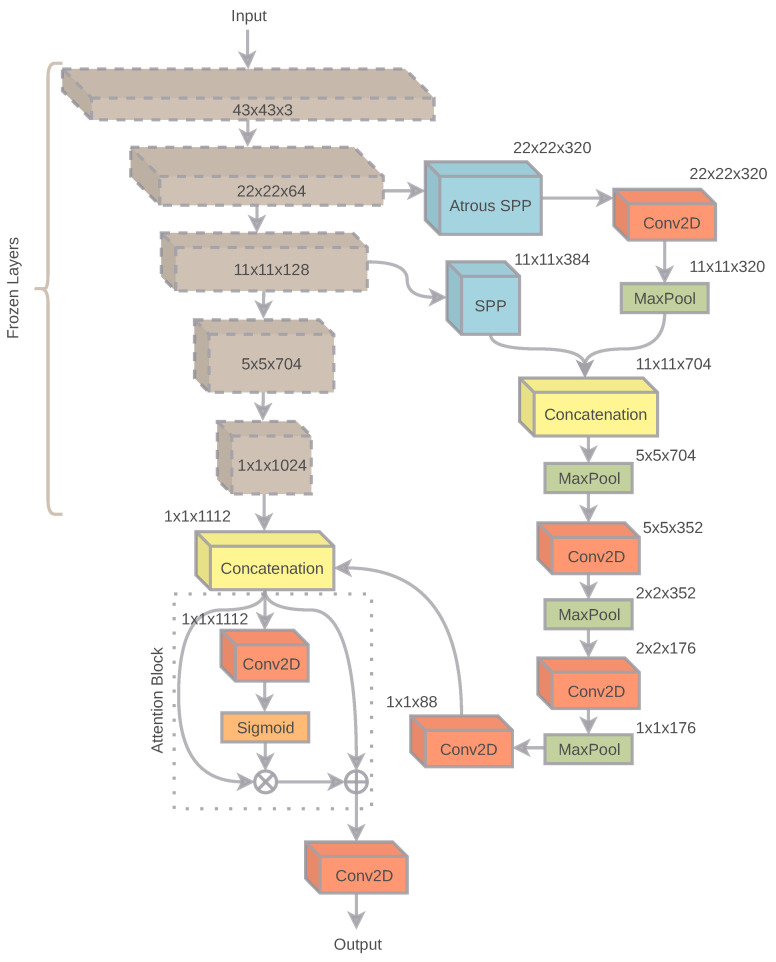
The figure explains the architecture of the embedding model. The given input (image patch) is passed through a frozen DCNN backend. Parallelly, ASPP and SPP blocks operate from two output dimensions (22×22×64 and 11×11×128, respectively) of the DCNN backend. The outputs of ASPP and SPP are concatenated, convolved, and finally merged with the DCNN backend’s output. The output is followed by a small attention block and generates final patch-based descriptors.

Figure 4 describes the architecture of the spatial network. Both features extracted by the SPP and ASPP are further concatenated and downsampled to a high-dimensional single-pixel feature. Successively, the outputs produced by the DCNN backbone and SPP+ASPP downsampled features are merged. The output of the merged features is passed through an attention block, which regulates the output sensitivity of the model [50]. The attention layer is followed by a final convolution layer of 64 kernels, which produces the final output by the spatial network. The convolutions conducted in the spatial network are conducted in the following pattern of activation, batch-normalization, and convolution, respectively. Excluding the DCNN baseline, the overall embedding model consists of 7,118,864 parameters.

#### 3.2.3. Network Training

**Figure 5:**
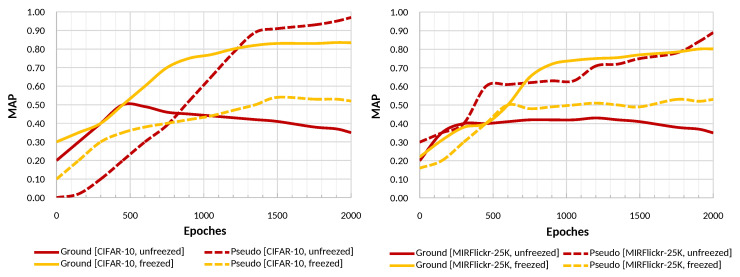
The figure illustrates a comparison of training the embedding model while keeping the DCNN backbone frozen and unfrozen. The left and right graphs are for CIFAR-10 and MIRFlickr-25K, sequentially. Pseudo and ground represent the training on pseudo labels and actual labels (given in the dataset), respectively.

The training of AutoRet is conducted using mean-square-error loss along with Adam [51] optimizer. In general, the architecture requires a minimum of 2000 epochs to converge to the optimal. While training, the pre-trained weights of the DCNN backbone inside the spatial network (explained in Section 3.2.2) are not updated. Updating the weights of the DCNN backbone causes the overall model to overfit on the pseudo-label, ignoring the ground/actual relationships. Figure 5 shows a comparison of training records keeping the DCNN backbone weights freezed (not updated) and unfreezed (updated).

## 4. Experiment

In this section, we present the datasets used in the evaluation process. Further, the metrics used for evaluation are discussed, followed by enlisting the candidate models. Finally, this section represents a comparison benchmark based on performance on different dimensions.

### 4.1. Dataset

Three datasets have been used to conduct the evaluation. One popular classification dataset and CIFAR-10 [52] is used in the evaluation. Further, two multi-class datasets, MIRFlickr-25K [53] and NUS-WIDE [54] have been used for benchmarking. Table 1 contains a quantitative detail of the datasets.

### 4.2. Evaluation Metrics

To evaluate the efficiency of the competing models, the following evaluation metrics are employed:

- **Mean Average Precision (MAP):** MAP is the most popular metric used to evaluate the performance of retrieval systems. The metric works by calculating the ranking of the accurately selected results, defined by:
  (4) $$\begin{array}{c} MAP=\frac{1}{Q}\sum_{i}^{\left|Q\right|}\left(\frac{1}{r}\sum_{j}^{r}p\left(i,j\right)\right) \end{array}$$
  Here |Q| is the size of the query set in which *r* is the number of correct returned images. p(i,j) represents the precision of *j*’th correct image over the *i*’th query image.
- **Precision/Recall @ N:** The metric describes the precision and recall rate based on the number of retrieved image samples (N) as threshold. In general, the correct retrieved images would appear early for a set of retrieved images. Therefore, the precision/recall result for a lower number of retrieved images is important than higher values of *N*.

### 4.3. Evaluation Baselines

We compare our model with the following un/self-supervised models: sparse graph based self-supervised hashing (SGSH) [18], self-supervised product quantization (SPQ) [14], deep variational binaries (DVB) [55], distillhash [17], binary generative adversarial networks (BGAN) [31], BinGAN [56], unsupervised deep hashing with pseudo labels (UDHP) [20], similarity adapdive deep hashing (SADH) [16]. For the hash-based methods, the number of bits is kept to 64 for best results. For AutoRet, the default output dimension is kept to be 16. The input image shape for all the datasets and models is kept to be 128×128. Therefore, images smaller and larger than 128×128 are re-adjusted.

### 4.4. Comparison

In the comparison, we foremost evaluate the AutoRet architecture with two different backbones: DenseNet121 [57] and MobileNet [58] which are observed to be implemented in the AutoEmbedder framework [48,49]. Table 2 depicts a comparison of AutoRet architecture with two distinct backbones. Moreover, benchmarks are also conducted with and without the SPP and ASPP mechanisms. The comparison explains that DenseNet121 performs better than the MobileNet framework. Further, adding SPP in both baselines greatly improves the retrieval performance. In the case of ASPP, the margin of improvement on CIFAR-10 tends to be higher than the SPP mechanism. CIFAR-10 contains low-resolution images up-sampled to 128×128 to feed the network. As ASPP provides an improved field of view, it enables better confidence for low-resolution images. Therefore, it can be concluded that ASPP performs better for low-resolution images. Consequently, fusing ASPP and SPP with the baselines improves the query performance.

Table 3 exhibits a comparison of AutoRet with different models based on MAP. By examining the table, it can be noticed that GAN-based architectures mostly perform marginally. In contrast, the graph-based self-supervised model SGSH performs better in MIRFlickr-25K and performs inadequately in the other datasets. SGSH is based on a sparse graph; therefore, it only receives strong edges, rejecting the less important but useful connections. As a result, SGSH suffers from graph connectivity issues. Parallelly, SPQ uses contrastive loss [59] in model training. However, SPQ misses to properly aggregate the overall description of an image. Therefore, the system can be misled by the background of an image. Comparatively, AutoRet achieves a better margin of improvement than SPQ due to better feature localization, resolution-independent feature extraction, and reasonable feature aggregation.

**Figure 6:**
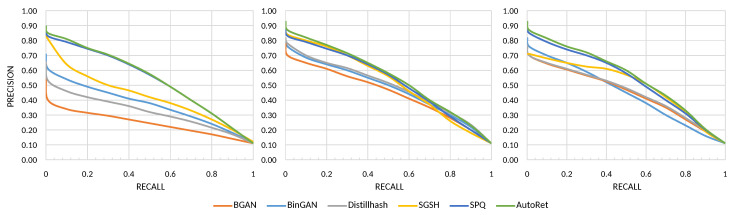
The figures illustrate precision-recall graphs measured on CIFAR-10, MIRFlickr-25K, and NUS-WIDE, respectively.

Figure 6 illustrates a precision-recall graph for some of the models in the benchmark. In the case of a precision-recall metric, SPQ offers to be a strong candidate with AutoRet. Although the SPQ and AutoRet perform similarly on CIFAR-10, AutoRet performs more promising than SPQ on other datasets.

AutoRet architecture can be mixed with labeled data while training in self-supervised strategy. The AutoEmbedder framework is generally trained with augmented and randomly selected data for can-link and cannot-link constraints, respectively. If some labeled data is added in the self-supervised policy, the can and cannot-link pairs can be correctly guessed without any augmentation and random selection process. Therefore, adding some labeled data has a great probability of improving the model’s performance.

**Figure 7:**
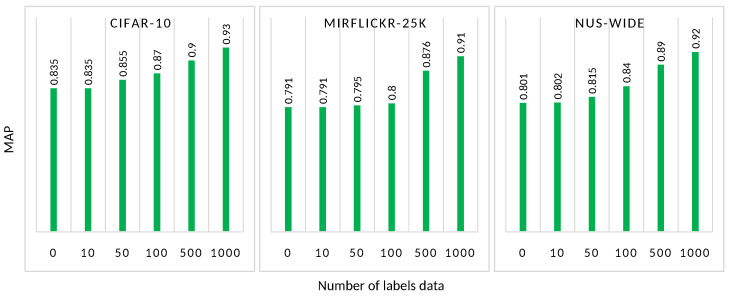
The figure represents the increase of MAP by mixing a small portion of labeled/ground data while training AutoRet. The three graphs exhibit the benchmark conducted in CIFAR-10, MIRFlickr-25K, and NUS-WIDE, respectively.

Figure 7 exhibits a benchmark of the AutoRet system while adding a small number of labeled data in the training strategy. Adding a small number of data (up to 50 known data samples) slightly improves the MAP score of the models. Further, increasing the number of labeled data samples boosts the MAP score of the model. Typically, adding at least 100 labeled samples starts to increase the performance of the model. Therefore AutoRet is a promising model that can work in both self-supervised as well as semi-supervised mode, based on the availability of labeled data.

**Figure 8:**
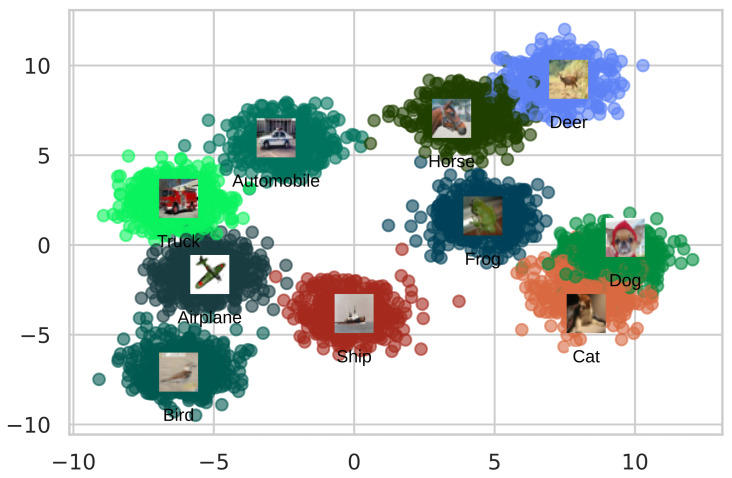
The figure illustrates a scatter plot of the embedding space generated on CIFAR-10 dataset.

Figure 8 depicts an embedding space generated by the AutoRet. The embeddings are reduced using t-SNE [60]. The embedding space illustrates strongly correlated clusters except for some outliers. The clusters of cat and dog have considerable overlap as both animals have visual similarities. In addition, horse and deer classes have similar outcomes. The rest of the class embeddings have a good cluster margin, excluding some anomalies.

**Figure 9:**
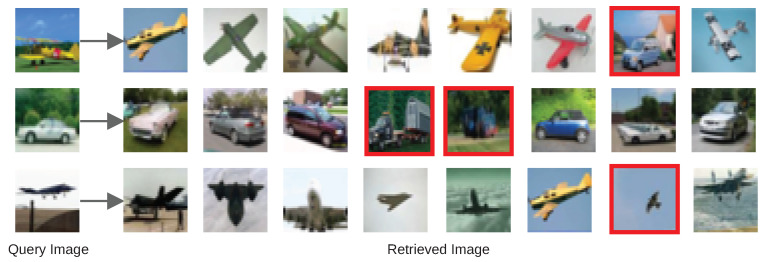
The figure depicts an inference of the query and the retrieved images. Each row represents a query and the corresponding retrieved images. Red bounded images are faulty retrievals.

Figure 9 further shows three inference examples with some faulty retrieval of the AutoRet. AutoRet focuses on local descriptors. Therefore, the wrong outputs contain high local similarities based on the query images. For the first query, the image contains terrain, sky, landscape, and a plane. In contrast, the faulty retrieved image contains terrain, sky, landscape, and an automobile. Due to the fusion of local descriptors, the incorrect retrievals are partially similar to the context of the image. Hence, the retrieval system is often contextually correct.

## 5. Conclusions

The paper proposes an image retrieval system, AutoRet, which can establish image relationships based on image content. The model is constructed with a spatial pooling based DCNN architecture, extracting high-quality embeddings from multiple portions of an image. Further, a recurrent neural network relates the embeddings and outputs prominent content information of a given image. The local feature extraction based on the spatial architecture is trained in a self-supervised manner, which can also utilize labeled data. We evaluate the model in three different datasets and determine that the proposed AutoRet performs competently in self-supervised training. Moreover, mixing a small portion of labeled data also improves the robustness of the model. Benchmarks evaluate that, AutoRet is competitive in performance on self-supervised learning in all of the datasets. Further, the performance of AutoRet is also prominent concerning the small increase in the number of labeled classes during the self-supervised training process. We strongly believe that this work would motivate researchers to invest endeavor in robust self-supervised based image retrieval systems, focusing on labeled data as well.

## Figures and Tables

**Table 1:** Quantitative analysis of the datasets.

Dataset	Classes	Data
CIFAR-10 [52]	10	60,000
MIRFlickr-25K [53]	38	25,000
NUS-WIDE [54]	80	270,000

**Table 2:** A comparison of different architectural constructions of the AutoRet.

Method	CIFAR-10 (MAP)	MIRFlickr-25K (MAP)	NUS-WIDE (MAP)
MobileNet	0.682	0.671	0.582
MobileNet+SPP	0.742	0.751	0.766
MobileNet+ASPP	0.767	0.764	0.768
MobileNet+SPP+ASPP	0.796	0.771	0.785
DenseNet121	0.716	0.688	0.610
DenseNet121+SPP	0.781	0.782	0.785
DenseNet121+ASPP	0.792	0.774	0.748
**DenseNet121+SPP+ASPP**	0.835	0.791	0.801

**Table 3:** A comparison of different image retrieval systems.

Method	CIFAR-10 (MAP)	MIRFlickr-25K (MAP)	NUS-WIDE (MAP)
SADH	0.377	0.481	0.563
BGAN	0.562	0.695	0.730
BinGAN	0.520	0.688	0.713
UDHP	0.384	0.680	0.526
Distillhash	0.287	0.708	0.621
DVB	0.396	0.524	0.595
SPQ	0.812	0.778	0.785
SGSH	0.469	0.739	0.628
**AutoRet (ours)**	0.835	0.791	0.801

## Data Availability

Data sharing not applicable.

## Abbreviations

The following abbreviations are used in this manuscript:

DCNN	Deep Convolutional Neural Network
CBIR	Content-based Image Retrieval
CNN	Convolutional Neural Network
GCNN	Graph Convolutional Neural Networks
SPP	Spatial Pyramid Convolution
ASPP	Atrous Spatial Pyramid Convolution
SRN	Spatial Recurrent Network
Bi-LSTM	Bi-directional Long Short-term Memory
MAP	Mean Average Precision
SGSH	Sparse Graph-based Self-supervised Hashing
SPQ	Self-supervised Product Quantization
DVB	Deep Variational Binaries
BGAN	Binary Generative Adversarial Networks
UDHP	Unsupervised Deep Hashing with Pseudo-labels
SADH	Similarity Adapdive Deep Hashing

## References

[1] Wang J., Hua X.S. (2011). Interactive image search by color map. ACM Trans. Intell. Syst. Technol. (TIST).

[2] Wang X.Y., Zhang B.B., Yang H.Y. (2014). Content-based image retrieval by integrating color and texture features. Multimed. Tools Appl..

[3] Bai S., Bai X., Zhou Z., Zhang Z., Jan Latecki L. Gift: A real-time and scalable 3d shape search engine. Proceedings of the IEEE Conference on Computer Vision and Pattern Recognition.

[4] Li Y., Shapiro L., Bilmes J.A. A generative/discriminative learning algorithm for image classification. Proceedings of the Tenth IEEE International Conference on Computer Vision (ICCV’05) Volume 1.

[5] Gudivada V.N., Raghavan V.V. (1995). Content based image retrieval systems. Computer.

[6] Wu Z., Ke Q., Sun J., Shum H.Y. (2011). Scalable face image retrieval with identity-based quantization and multireference reranking. IEEE Trans. Pattern Anal. Mach. Intell..

[7] Feng F., Niu T., Li R., Wang X., Jiang H. Learning Visual Features from Product Title for Image Retrieval. Proceedings of the 28th ACM International Conference on Multimedia.

[8] Deng D., Wang R., Wu H., He H., Li Q., Luo X. (2018). Learning deep similarity models with focus ranking for fabric image retrieval. Image Vis. Comput..

[9] Huang J., Feris R.S., Chen Q., Yan S. Cross-domain image retrieval with a dual attribute-aware ranking network. Proceedings of the IEEE International Conference on Computer Vision.

[10] Qayyum A., Anwar S.M., Awais M., Majid M. (2017). Medical image retrieval using deep convolutional neural network. Neurocomputing.

[11] Li X., Yang J., Ma J. (2021). Recent developments of content-based image retrieval (CBIR). Neurocomputing.

[12] Wang Z., Liu X., Li H., Shi J., Rao Y. (2018). A Saliency Detection Based Unsupervised Commodity Object Retrieval Scheme. IEEE Access.

[13] Shin M., Park S., Kim T. (2019). Semi-supervised feature-level attribute manipulation for fashion image retrieval. arXiv.

[14] Jang Y.K., Cho N.I. Self-supervised Product Quantization for Deep Unsupervised Image Retrieval. Proceedings of the IEEE/CVF International Conference on Computer Vision.

[15] Doersch C., Zisserman A. Multi-task self-supervised visual learning. Proceedings of the IEEE International Conference on Computer Vision.

[16] Shen F., Xu Y., Liu L., Yang Y., Huang Z., Shen H.T. (2018). Unsupervised deep hashing with similarity-adaptive and discrete optimization. IEEE Trans. Pattern Anal. Mach. Intell..

[17] Yang E., Liu T., Deng C., Liu W., Tao D. Distillhash: Unsupervised deep hashing by distilling data pairs. Proceedings of the IEEE/CVF Conference on Computer Vision and Pattern Recognition.

[18] Wang W., Zhang H., Zhang Z., Liu L., Shao L. (2021). Sparse graph based self-supervised hashing for scalable image retrieval. Inf. Sci..

[19] Kaya M., Bilge H.Ş. (2019). Deep metric learning: A survey. Symmetry.

[20] Zhang H., Liu L., Long Y., Shao L. (2017). Unsupervised deep hashing with pseudo labels for scalable image retrieval. IEEE Trans. Image Process..

[21] Buslaev A., Iglovikov V.I., Khvedchenya E., Parinov A., Druzhinin M., Kalinin A.A. (2020). Albumentations: Fast and flexible image augmentations. Information.

[22] Andriyanov N. (2021). Methods for preventing visual attacks in convolutional neural networks based on data discard and dimensionality reduction. Appl. Sci..

[23] Vizilter Y.V., Vygolov O., Zheltov S.Y. (2021). Morphological analysis of mosaic shapes with directed relationships based on attribute and relational model representations. Comput. Opt..

[24] Vasil’ev K.K., Dement’ev V.E., Andriyanov N.A. (2015). Doubly stochastic models of images. Pattern Recognit. Image Anal..

[25] Chaplot D.S., Dalal M., Gupta S., Malik J., Salakhutdinov R.R. (2021). SEAL: Self-supervised Embodied Active Learning using Exploration and 3D Consistency. Adv. Neural Inf. Process. Syst..

[26] Tsai Y.H.H., Wu Y., Salakhutdinov R., Morency L.P. (2020). Self-supervised learning from a multi-view perspective. arXiv.

[27] Yang X., Qian X., Mei T. (2015). Learning salient visual word for scalable mobile image retrieval. Pattern Recognit..

[28] Lin G., Shen C., Shi Q., Van den Hengel A., Suter D. Fast supervised hashing with decision trees for high-dimensional data. Proceedings of the IEEE Conference on Computer Vision and Pattern Recognition.

[29] Liu H., Ji R., Wu Y., Liu W. Towards optimal binary code learning via ordinal embedding. Proceedings of the AAAI Conference on Artificial Intelligence.

[30] Zhou X., Shen F., Liu L., Liu W., Nie L., Yang Y., Shen H.T. (2018). Graph convolutional network hashing. IEEE Trans. Cybern..

[31] Song J., He T., Gao L., Xu X., Hanjalic A., Shen H.T. Binary generative adversarial networks for image retrieval. Proceedings of the Thirty-second AAAI conference on artificial intelligence.

[32] Cao Y., Long M., Wang J., Liu S. Deep visual-semantic quantization for efficient image retrieval. Proceedings of the IEEE Conference on Computer Vision and Pattern Recognition.

[33] Lin K., Lu J., Chen C.S., Zhou J. Learning compact binary descriptors with unsupervised deep neural networks. Proceedings of the IEEE Conference on Computer Vision and Pattern Recognition.

[34] Erin Liong V., Lu J., Wang G., Moulin P., Zhou J. Deep hashing for compact binary codes learning. Proceedings of the IEEE Conference on Computer Vision and Pattern Recognition.

[35] Shen J., Tang X., Dong X., Shao L. (2019). Visual object tracking by hierarchical attention siamese network. IEEE Trans. Cybern..

[36] He X., Zhou Y., Zhou Z., Bai S., Bai X. Triplet-center loss for multi-view 3d object retrieval. Proceedings of the IEEE Conference on Computer Vision and Pattern Recognition.

[37] Kim S., Seo M., Laptev I., Cho M., Kwak S. Deep metric learning beyond binary supervision. Proceedings of the IEEE/CVF Conference on Computer Vision and Pattern Recognition.

[38] Sharif Razavian A., Azizpour H., Sullivan J., Carlsson S. CNN features off-the-shelf: An astounding baseline for recognition. Proceedings of the IEEE Conference on Computer Vision and Pattern Recognition.

[39] Paulin M., Douze M., Harchaoui Z., Mairal J., Perronin F., Schmid C. Local convolutional features with unsupervised training for image retrieval. Proceedings of the IEEE International Conference on Computer Vision.

[40] Wei X.S., Luo J.H., Wu J., Zhou Z.H. (2017). Selective convolutional descriptor aggregation for fine-grained image retrieval. IEEE Trans. Image Process..

[41] Babenko A., Lempitsky V. Aggregating local deep features for image retrieval. Proceedings of the IEEE International Conference on Computer Vision.

[42] Xu J., Shi C., Qi C., Wang C., Xiao B. Unsupervised part-based weighting aggregation of deep convolutional features for image retrieval. Proceedings of the AAAI Conference on Artificial Intelligence.

[43] Arandjelovic R., Gronat P., Torii A., Pajdla T., Sivic J. NetVLAD: CNN architecture for weakly supervised place recognition. Proceedings of the IEEE Conference on Computer Vision and Pattern Recognition.

[44] He K., Zhang X., Ren S., Sun J. (2015). Spatial pyramid pooling in deep convolutional networks for visual recognition. IEEE Trans. Pattern Anal. Mach. Intell..

[45] Chen L.C., Papandreou G., Kokkinos I., Murphy K., Yuille A.L. (2017). Deeplab: Semantic image segmentation with deep convolutional nets, atrous convolution, and fully connected crfs. IEEE Trans. Pattern Anal. Mach. Intell..

[46] Zhang Z., Liu L., Shen F., Shen H.T., Shao L. (2018). Binary multi-view clustering. IEEE Trans. Pattern Anal. Mach. Intell..

[47] Gu Y., Wang S., Zhang H., Yao Y., Yang W., Liu L. (2019). Clustering-driven unsupervised deep hashing for image retrieval. Neurocomputing.

[48] Ohi A.Q., Mridha M.F., Safir F.B., Hamid M.A., Monowar M.M. (2020). Autoembedder: A semi-supervised DNN embedding system for clustering. Knowl.-Based Syst..

[49] Mridha M.F., Ohi A.Q., Monowar M.M., Hamid M.A., Islam M.R., Watanobe Y. (2021). U-Vectors: Generating Clusterable Speaker Embedding from Unlabeled Data. Appl. Sci..

[50] Kateb F.A., Monowar M.M., Hamid M., Ohi A.Q., Mridha M.F. (2021). FruitDet: Attentive Feature Aggregation for Real-Time Fruit Detection in Orchards. Agronomy.

[51] Kingma D.P., Ba J. (2014). Adam: A method for stochastic optimization. arXiv.

[52] Krizhevsky A., Hinton G. (2009). Learning Multiple Layers of Features from Tiny Images. https://www.cs.toronto.edu/kriz/learning-features-2009-TR.pdf.

[53] Huiskes M.J., Lew M.S. The mir flickr retrieval evaluation. Proceedings of the 1st ACM International Conference on Multimedia Information Retrieval.

[54] Chua T.S., Tang J., Hong R., Li H., Luo Z., Zheng Y. Nus-wide: A real-world web image database from national university of singapore. Proceedings of the ACM International Conference on Image and Video Retrieval.

[55] Shen Y., Liu L., Shao L. (2019). Unsupervised binary representation learning with deep variational networks. Int. J. Comput. Vis..

[56] Zieba M., Semberecki P., El-Gaaly T., Trzcinski T. (2018). Bingan: Learning compact binary descriptors with a regularized gan. arXiv.

[57] Huang G., Liu Z., Van Der Maaten L., Weinberger K.Q. Densely connected convolutional networks. Proceedings of the IEEE Conference on Computer Vision and Pattern Recognition.

[58] Howard A.G., Zhu M., Chen B., Kalenichenko D., Wang W., Weyand T., Andreetto M., Adam H. (2017). Mobilenets: Efficient convolutional neural networks for mobile vision applications. arXiv.

[59] Khosla P., Teterwak P., Wang C., Sarna A., Tian Y., Isola P., Maschinot A., Liu C., Krishnan D. (2020). Supervised contrastive learning. arXiv.

[60] Van der Maaten L., Hinton G. (2008). Visualizing data using t-SNE. J. Mach. Learn. Res..

